# Unexpected high frequency of neurofibroma in the celiac ganglion of German cattle

**DOI:** 10.1186/s13567-020-00800-1

**Published:** 2020-06-17

**Authors:** Insa Dammann, Wiebke M. Wemheuer, Arne Wrede, Wilhelm E. Wemheuer, Amely Campe, Jutta Petschenka, Ulf Schulze-Sturm, Uwe Hahmann, Claus P. Czerny, Pia Münster, Bertram Brening, Lothar Kreienbrock, Christiane Herden, Walter J. Schulz-Schaeffer

**Affiliations:** 1 Institute of Neuropathology, Medical Faculty of the Saarland University, Homburg, Germany; 2 Institute of Neuropathology, University Medical Center Goettingen, Göttingen, Germany; 3 Institute of Veterinary Medicine, University of Goettingen, Göttingen, Germany; 4 Department for Biometry, Epidemiology and Information Processing (IBEI), University of Veterinary Medicine and WHO-Collaboration Centre for Research and Training at the Human-Animal-Environmental Interface, Hannover, Germany; 5 Institute of Pathology, Veterinary Faculty, Justus Liebig University, Gießen, Germany; 6 Landeslabor Schleswig Holstein, Geschäftsbereich 2 Veterinärwesen, Neumünster, Germany; 7 Boehringer Ingelheim Pharma GmbH & Co. KG, Cancer Immunology & Immune Modulation, Biberach an der Riss, Germany; 8 Department of Paediatrics, University Medical Centre Hamburg-Eppendorf (UKE), Hamburg, Germany; 9 Elanco Deutschland GmbH, Hauptsitz Werner-Reimers-Str. 2-4, Bad Homburg, Germany

## Abstract

In a study originally designed to find potential risk factors for bovine spongiform encephalopathy (BSE) we examined tissues from 403 Holstein Frisian cattle in total. These included 20 BSE cattle and their 236 birth- and feeding cohort animals plus 32 offspring, 103 age, breed and district-matched control cattle and further twelve cattle with neurological signs. In addition to the obex, we examined the celiac ganglion, cervical cranial ganglion, trigeminal ganglion and proximal ganglion of the vagus nerve using histological techniques. Unexpectedly, we found a high number of neurofibroma, a benign peripheral nerve sheath tumor consisting of Schwann cells, fibroblasts and perineural cells. The neurofibroma were present only in the celiac ganglion and found during histologic examination. With a frequency of 9.91% in BSE cattle and their cohorts (case animals) and 9.09% in the age, breed and district matched control animals there seems to be no correlation between the occurrence of BSE and neurofibroma. Benign peripheral nerve sheath tumors have been described more often in cattle than in other domestic animals. Usually, they are incidental macroscopic findings in the thoracic ganglia during meat inspection. To our knowledge, there are no previous systematic histologic studies including bovine celiac ganglia at all. The high incidence of celiac ganglia neurofibroma may play a role in the frequently occurring abomasal displacements in Holstein Frisian cattle as the tumors might cause a gastrointestinal motility disorder. At present a genetic predisposition for these neoplasms cannot be ruled out.

## Introduction

Systematic sampling of nerve tissues in asymptomatic dairy cattle and their histologic investigation are rare, even though they give valuable information on animal health and the epidemiologic status of infectious diseases such as Listeriosis [1, 2]. In the present study, we had the unique opportunity to examine nerve tissues from a total of 403 German cattle in a setting that was planned and taken out during the BSE crisis. Cases of bovine spongiform encephalopathy (BSE) in Germany initiated a project in which the acquisition of samples from BSE cohort cattle was planned to find potential risk factors promoting prion propagation. For this purpose, also a selection of extra-cerebral tissues was made on the basis of experimental studies in different prion diseases in which pathological prion protein was detected before it reached the brainstem [3–5]. We chose the celiac ganglion, cervical cranial ganglion, trigeminal ganglion and the proximal ganglion of the vagus nerve designated as nodose ganglion according to previous studies. As controls served cattle that were matched regarding age, district and breed and had been slaughtered for human consumption. Breed (Holstein Frisians or Holstein Red) and the mean age of the case animals (73.7 ± 12.6 months), i.e. BSE cases and their birth- and feeding cohorts, and the control group (74.9 ± 13.9 months) mirror the circumstances under which the samples were acquired: In the North of Germany, Holstein Frisian is the prevailing breed for milk production and cattle with classic BSE usually fall sick or develop detectable prion protein aggregates in the obex region between 3 and 5 years of age.

Among the expected secondary findings were also neoplastic changes, especially benign peripheral nerve sheath tumors. Mostly, bovine tumors of the peripheral and central nervous system are not found in asymptomatic cattle [1, 2], but in cattle with neurological signs; among these, neoplasia are detectable with a percentage of approximately 2–7%; [6–9]. The occurrence of astrocytoma, ependymoma, glioblastoma multiforme, medulloblastoma, fibroma, bovine leucosis, bovine neurofibromatosis and malignant peripheral nerve sheath tumors have been described [6–9]. Benign peripheral nerve sheath tumors represent with 8–14% the most common kind of bovine tumor in Europe, Australia and North America [10–12]. In contrast to most of the above-mentioned types of tumor, they are incidental macroscopic findings during meat inspection located in ganglia of the heart, paravertebral ganglia of thorax and mediastinum, intercostal nerves and parts of the plexus brachialis. Interestingly, cattle seem to have benign peripheral nerve sheath tumors more often than other domestic animals [13]. The group of benign peripheral nerve sheath tumors classically includes schwannoma, neurofibroma and perineurioma. These tumors do not necessarily cause symptoms, but their growth may lead to a loss of function of the affected nerves or ganglia. Studies on macroscopically detected bovine peripheral nerve sheath tumors in Danish abattoirs revealed during histologic examination that the majority were in fact schwannoma, neurofibroma or a hybrid between those two benign tumor types [14, 15]. Malignant peripheral nerve sheath tumors represented less than 8% of all peripheral nerve sheath tumors and could be distinguished from their benign counterparts by widespread necrosis and hemorrhage, marked pleomorphism, nuclear atypia and the loss of differentiated Schwann cell markers such as S100 and CNPase [14]. In other species, e.g. dogs, the expression of the p75 neurotrophin receptor (p75^NTR^) for immature and non-myelinating Schwann cells proved to be a useful marker for the discrimination of benign peripheral nerve sheath tumors from other spindeloid tumors [16]. For a special peripheral nerve sheath neoplasm of Schwann cell origin, the so-called Devil facial tumor disease (DFTD) in Tasmanian devils, periaxin as a protein expressed by myelinating Schwann cells has become the most useful marker to confirm its presence [17]. Intriguingly, the thorough histological examination of peripheral nerve tissues in this study revealed neoplastic changes only in the celiac ganglion. Histopathology and immunohistochemistry helped to identify the tumors as neurofibroma, which consist mainly of Schwann cells and fibroblasts and sometimes perineural cells. In the article, we focus on the diagnosis of these tumors and discuss the potential meaning of their unexpected high incidence.

## Materials and methods

### Animals

The original study was designed to find early stages of bovine spongiform encephalopathy (BSE) and potential risk factors for BSE in the birth- and feeding cohorts of BSE animals according to Regulation (EC) No 999/2001. The choice of tissue samples had been made according to the experimentally proven route of spread of this prion disease after ingestion of the infectious agent [3–5]. Ganglia from 403 cattle were analyzed for the present study. The celiac ganglion was available in necessary quality and quantity in 322 animals. These included four BSE cattle and 218 animals from birth- and feeding cohorts (cohort animals and BSE animals are summarized as case animals in the following) plus 25 offspring from BSE cases (referred to as offspring). A representative control group for the birth cohort animals by means of age, breed and region in Lower Saxony, Germany, was selected from cattle that were slaughtered for human consumption. In addition, the celiac ganglia from nine animals with neurological signs, that had been suspected to have BSE but proved to be free of prion disease post-mortem, were investigated (referred to as CNS symptomatic group). All cattle belonged to the Holstein–Friesian or Red Holstein breed, except for one animal from the CNS symptomatic group, which was a German Simmental × Holstein–Friesian crossbreed. The availability of all ganglia for examination for each group and single cohort are listed in Tables 1 and 2.

**Table 1:** **Availability of four different ganglia for histopathological examination.**

	Number of animals examined	Celiac ganglia	Trigeminal ganglia	Nodose ganglia	Cervical cranial ganglia
BSE cattle	20	4	14	11	10
Cohort animals	236	218	176	140	104
BSE cattle offspring	32	25	18	19	15
Control cattle	103	66	89	71	51
CNS symptomatic cattle	12	9	12	11	8
Total	403	322	309	252	188

**Table 2:** **Availability of four different ganglia for histopathological examination in each cohort.**

Cohort	Celiac ganglia	Trigeminal ganglia	Nodose ganglia	Cervical cranial ganglia
95/01	9	0	0	0
10/02	24	22	12	20
40/02	7	5	5	6
46/02	0	5	5	5
48/02	6	6	7	3
53/02	2	2	2	1
54/02	24	22	17	9
64/02	13	14	7	9
79/02	10	8	8	4
86/02	15	15	13	8
88/02	5	2	0	0
93/02	8	10	8	9
99/02	4	4	3	1
22/03	16	14	11	5
01/04	3	1	0	0
14/04	1	1	1	1
18/04	4	4	4	3
29/04	7	4	4	3
36/04	12	12	10	6
37/04	4	2	3	1
47/04	23	10	11	5
50/04	3	1	1	0
57/04	5	5	5	2
21/05	12	6	3	3
11/06	1	1	0	0
Total	218	176	140	104

### Sample acquisition

Animals were either euthanized (cohort animals, offspring CNS symptomatic group, BSE animals with symptoms) or slaughtered (control animals, BSE animals found in abattoirs). The obex region of each cohort animal and all slaughtered control animals underwent rapid testing in authorized laboratories (Lower Saxony State Office for Consumer Protection and Food safety (LAVES), Oldenburg, Germany and Institute of Veterinary Medicine, Göttingen, Germany) in accordance with the Regulation (EC) No 999/2001 (Appendix X, chapter C). In the following, the skull was opened and brain, both trigeminal ganglia and both proximal ganglia of the vagus nerve were extracted and dissected. The abdomen was cut open and the celiac ganglion including parts of the mesenterial artery and the celiac truncus were removed (cohort animals). For regularly slaughtered animals (controls) the celiac ganglion plus torn off blood vessels were usually obtained from the removed gastrointestinal tract, seldom from the carcass. The latter was not feasible for the regularly slaughtered BSE animals that were identified in the due course of rapid testing, since the gastrointestinal tract had been disposed of by that time.

### Histopathology

From each animal, the left trigeminal ganglion, left cervical cranial ganglion and the left proximal ganglion of the vagus nerve were fixed in 4% buffered formaldehyde (equals 10% buffered formalin) for at least 48 h while the corresponding ganglia from the right side were stored at −20 °C. The celiac ganglion was cut at right angle to the two blood vessels, which were left in place, and two slices were fixed in formaldehyde while the rest was stored at −20 °C.

The formalin-fixed tissues were embedded in paraffin according to a standard protocol. Decontamination with formic acid [18] was only performed in cases where the rapid test indicated the presence of pathological prion protein (BSE cases). One to 3 µm sections were cut and put on silane-coated glass slides or superfrost glass slides and stained with hematoxylin and eosin (H/E). For better depiction of the connective tissue, an Elastica-van-Gieson staining was performed and basal membranes were visualized using Gomori silver impregnation on all ganglia with neoplastic alterations.

### Immunohistochemistry

One to 3 µm thick paraffin sections were placed on superfrost glass slides and rehydrated for the antibody interaction using standard protocols. Sections were then pretreated with steps for antigen retrieval and blocking of background reactions. During microwave treatment the slides were boiled five times for 3 min at 200 W in a cuvette with citrate buffer (0.01 M citric acid, pH 6) in a commercially available microwave (NN-E201-WM, Panasonic). Boiling temperature was reached by 60-second intervals at decreasing wattage (800 W, 700 W, 360 W, 270 W, 200 W). Slides were left to cool for 30 min afterwards at room temperature. For steamer treatment, slides with 4 mM hydrochloric acid (HCl) were put into a commercially available kitchen steamer (Multigourmet type 3216, Braun GmbH) for a total of 45 min (including 15 min needed to reach the necessary temperature) and left to cool down at room temperature for 30 min afterwards. Blocking with 0.2% casein (I-Block, Applied Biosystems, USA) in PBS including 0.5% Tween (Carl Roth GmbH) or 3% fetal calf serum (FCS, PAA Laboratories GmbH) in TBS and the inactivation of endogenous peroxidases with H_2_O_2_ 3% or 0.1% H_2_O_2_ in methanol was done for 30 min each. These steps are summarized for each primary antibody in Table 3. Between all steps the sections were rinsed three times in Tris-buffered saline (TBS, 1 M Tris–HCl, 1.5 M NaCl, pH 7.8). Slides were incubated with the respective primary and secondary antibody in TBS for 60 min at room temperature if not indicated otherwise. The secondary antibody reactions were visualized using either New Fuchsine, 3-Amino-9-ethylcarbazol (AEC) or 3,3´-Diaminobenzidine (DAB) as a chromogens [19, 20]. After the chromogen reaction, sections were lightly counterstained with Hemalaun and coverslipped with Aquadex (Merck Millipore, MA, USA).

**Table 3:** **Primary antibodies used for immunohistochemistry and their respective pre-treatments, secondary antibodies and chromogens.**

Primary antibody	Dilution	Pre-treatments (antigen retrieval/blocking)	Secondary antibody	Dilution	Chromogen
S-100 (Z0311, Dako, Agilent, Waldbronn)	1:400	Citrate buffer in microwave/0.2% caseine	Anti-rabbit alkaline phosphatase (AP)-coupled (D0487, Dako, Agilent, Waldbronn)	1:50	New Fuchsine
Vimentin (M7020, Dako, Agilent, Waldbronn)	1:300	Protease 0.5% 30 min/0.2% casein, 3% H_2_O_2_	Anti-mouse biotinylated (RPN1001, GE Healthcare Europe GmbH, Freiburg)	1:100	AEC
GFAP (Z0334, Dako, Agilent, Waldbronn)	1:1000	−/0.2% casein	Anti-rabbit AP-coupled (see above)	1:50	New Fuchsine
Neurofilament (M0762, Dako, Agilent, Waldbronn)	1:100	−/0.2% casein	Anti-mouse alkaline phosphatase (AP)-coupled (D0486, Dako, Agilent, Waldbronn)	1:500	New Fuchsine
PGP 9.5 (PgP9.5-L-CE, Leica Biosystems, Nussloch)	1:20	Citrate buffer in microwave/0.2% casein, 3% H_2_O_2_	Anti-mouse biotinylated (see above)	1:100	AEC
MBP (A0623, Dako, Agilent, Waldbronn)	1:500, overnight 4 °C	−/3% FCS in TBS, 0.1% H_2_O_2_ in methanol 30 min	Biotinylated anti-rabbit (RPN1004, GE Healthcare Europe GmbH, Freiburg)	1:1000 30 min	Tertiary antibody: Strepatvidin—HRP (SN1004, Invitrogen, Thermo Fisher Scientific, USA) chromogen: DAB
Neurofibromin 1 (LS-B8110/49155, Biozol Diagnostica Vertrieb GmbH, Eching)	1:50, overnight 4 °C	HCl (4 mM) in steamer/0.2% casein, 3% H_2_O_2_	Biotinylated anti-rabbit (see above)	1:100	AEC
Neurofibromin 2 (sc-331, Santa Cruz Biotechnology, Inc., Heidelberg)	1:50, overnight 4 °C	HCl (4 mM) in steamer/0.2% casein/3% H_2_O_2_	Biotinylated anti-rabbit (see above)	1:100	AEC

Two investigators viewed blinded samples under a microscope (Olympus BX41) with 20 × , 40 × , 100 × and 400 × magnifications. Pictures were taken with a digital camera (Olympus DP71) attached to an Olympus BX51 microscope using the software CellSense Dimensions 1.7.1 (Olympus).

### Statistical analysis

The presence of neurofibroma was noted during histopathological analysis of the samples. When the investigators had been unblinded, and the obtained data was assigned to the respective groups, we applied Kruskall Wallis one-way analysis of variance using Graph Pad 6 software to elucidate if there were any significant differences between case animals (BSE animals and their cohorts) and control cattle regarding the number of animals affected by neurofibroma in the celiac ganglion. The percentage of all animals with neurofibroma per group (case animals and controls, but also BSE animals, cohorts, each single cohort, offspring, CNS symptomatic animals) was furthermore depicted using Graph pad 6 software. The percentage of cohort animals with neurofibroma is additionally shown as a dotted line. For a better overview of the study groups, the age of all groups was also depicted using Graph Pad 6 software.

Age of animals with and without neurofibroma was compared within the case animal group and the control group. Furthermore, case animals and control animals were put into age groups spanning 6 months and the percentage of cattle with neurofibroma in the celiac ganglion was calculated. Each group with at least five animals and ganglia suitable for analysis was included into linear regression analysis plotting age as well as the occurrence of neurofibroma.

## Results

### Detection and characterization of tumors in the celiac ganglion

All investigated ganglia were without evident macroscopic pathological findings. Some of the celiac ganglia showed small nodules when cut, which was not recorded as a pathological finding at that time.

Upon histopathological investigation, some of the available celiac ganglia showed differentiated tumors of mesenchymal origin that are described in more detail below. The tumor size in the histologically examined sections varied from a diameter of 2 mm to 18 × 8 mm. The exact numbers of neoplasms in analyzable celiac ganglia are given in Table 4.

**Table 4:** **The study groups including their mean age and occurrence of neurofibroma.**

	Number of all animals	Age in months of all animals	Number of analyzable celiac ganglia	Age in months animals with analyzable celiac ganglia	Number of animals with neuro-fibroma in the celiac ganglion	% of neuro-fibroma in analyzable celiac ganglia	Age in months of animals with neuro-fibroma
Case animals (BSE + cohorts)	256	73.7 ± 12.6	222	73.4 ± 12.7	22	9.91	73.7 ± 12.2
Control cattle	103	74.9 ± 13.9	66	74.4 ± 11.8	6	9.09	72.2 ± 3.4
BSE	20	78.5 ± 11.6	4	76.8 ± 11.5	1	25	82
Cohorts	236	73.2 ± 12.6	218	73.2 ± 12.8	21	9.63	73.3 ± 12.3
Offspring	32	17.3 ± 9.1	25	17.3 ± 7	0	0	–
CNS Symptomatic	12	68.7 ± 18.4	9	64 ± 18.7	2	22.22	64 ± 3

**Figure 1:**
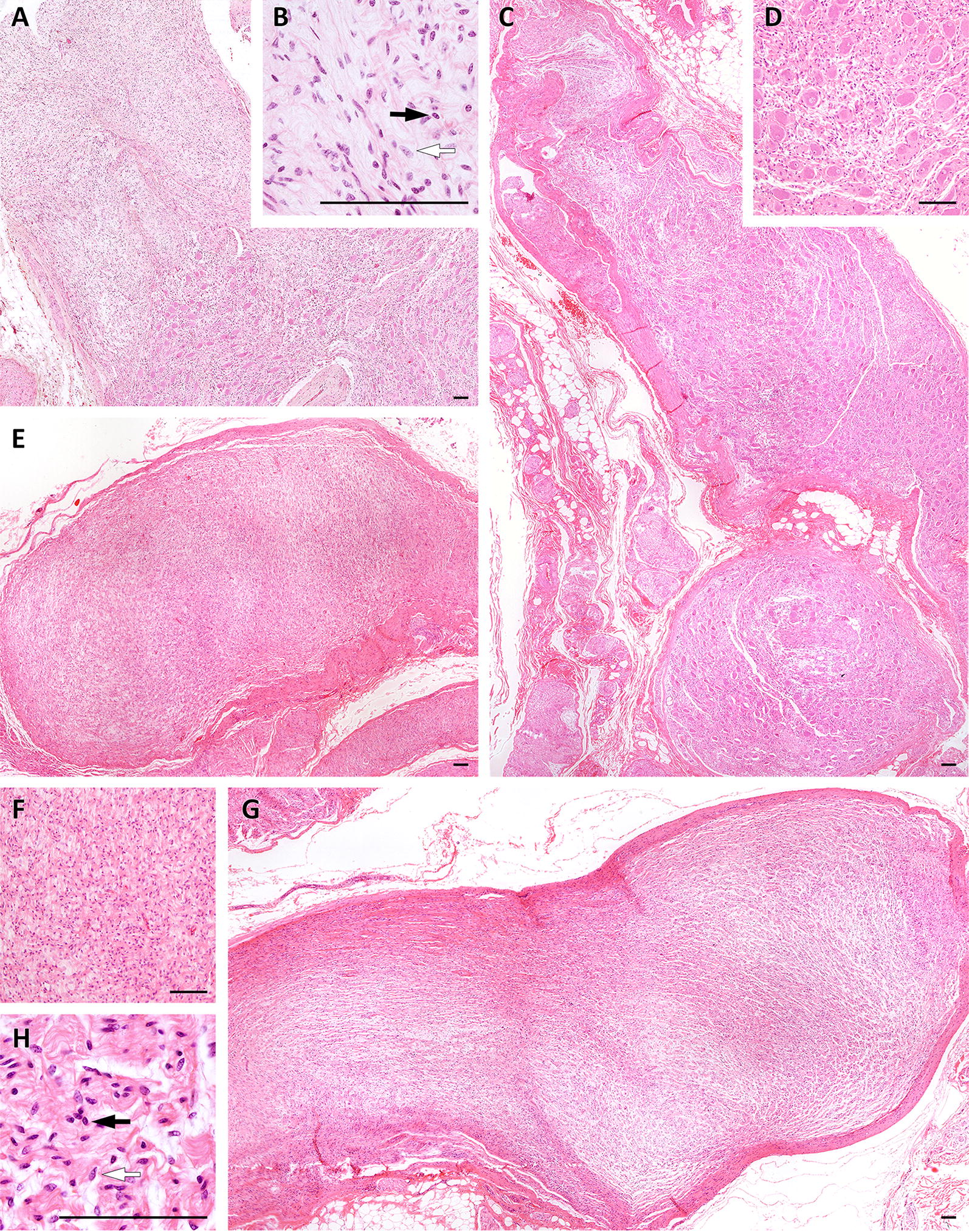
**Different growth patterns of neurofibroma in the bovine celiac ganglion**. **A** myxoid matrix with comparatively few cells next to infiltrated ganglion tissue is visible (**A**, **B**) showing two cell types: cells with small elongated nuclei with condensed chromatin (**B**, **H**; black arrows) and cells with large rather ovoid nuclei and coarse chromatin (**B**, **H** white arrows). Tumor cells infiltrating ganglion tissue can be observed (**C**, **D**) as well as compact neurofibroma with densely packed cells surrounded by a pseudo capsule formed by thickened perineurium (**E**, **F**) and lightly packed cells mimicking layers surrounded by a pseudo capsule (**G**, **H**). bar = 100 µm.

**Figure 2:**
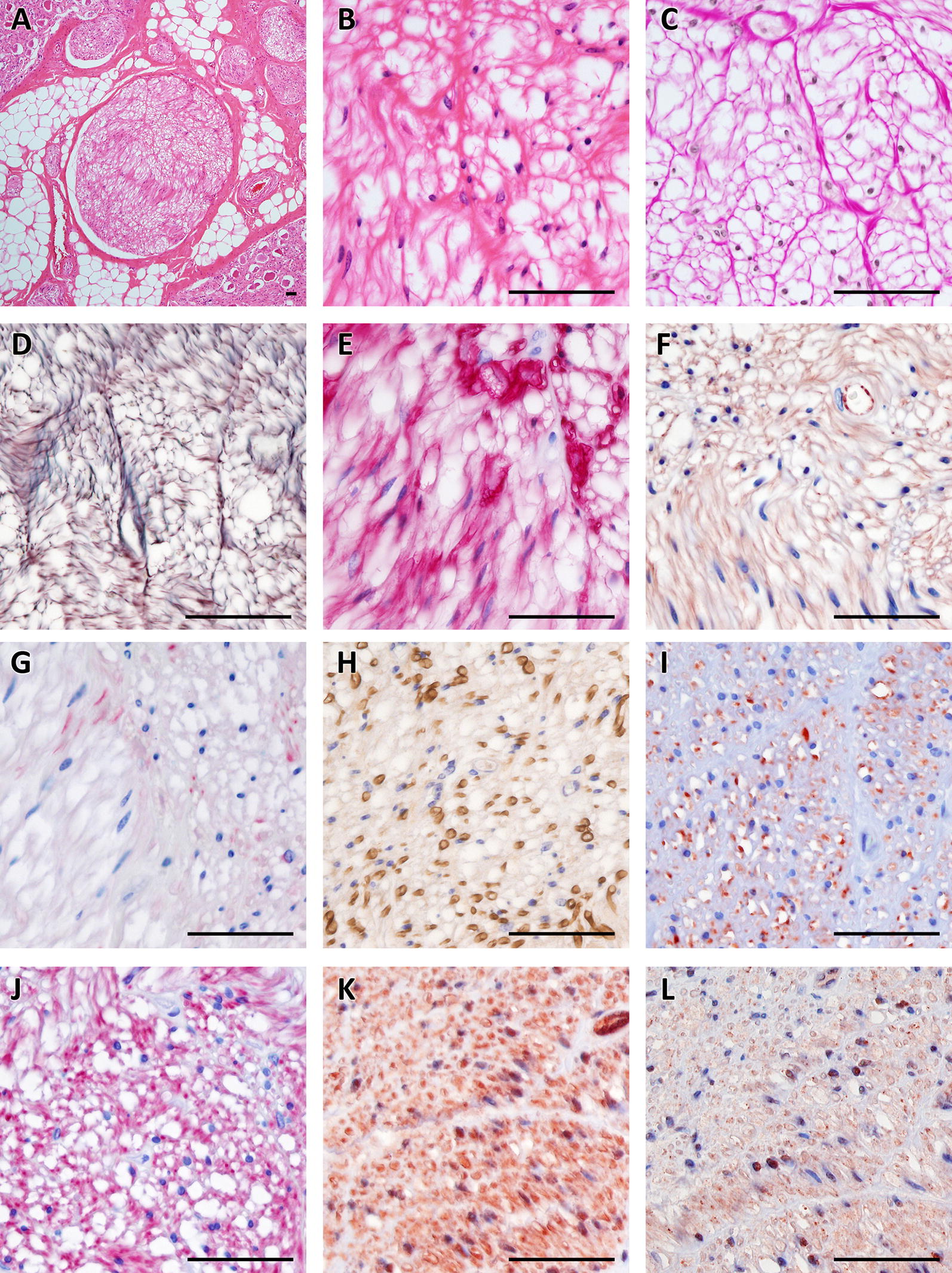
**Healthy peripheral nerve tissue**. Hematoxylin/Eosin staining shows a bundle of nerve fibers ensheathed by the perineurium and surrounded by fat tissue and further nerve fascicles at 40 × magnification (**A**). At 400x magnification the structure of the endoneurium (**B**) is visible. Elastica-van-Gieson staining (**C**) and silver stain according to Gomori (**D**) reveal the content of collagen and elastic fibers of the endoneurium. In the peripheral nerve system Schwann cells produce the myelin nerve sheaths; they express S100 (**E**), Vimentin (**F**) and at a low level GFAP (**G**). Axons are visualized by Neurofilament (**J**) and PGP9.5 (**I**), and myelinated nerve fibers by MBP (**H**). Neurofibromin 1 (**K**) is detectable in the cytoplasm and nuclei of Schwann cells while Neurofibromin 2 is mainly found by immunohistochemistry in the nucleus (**L**). bar = 50 µm.

**Figure 3:**
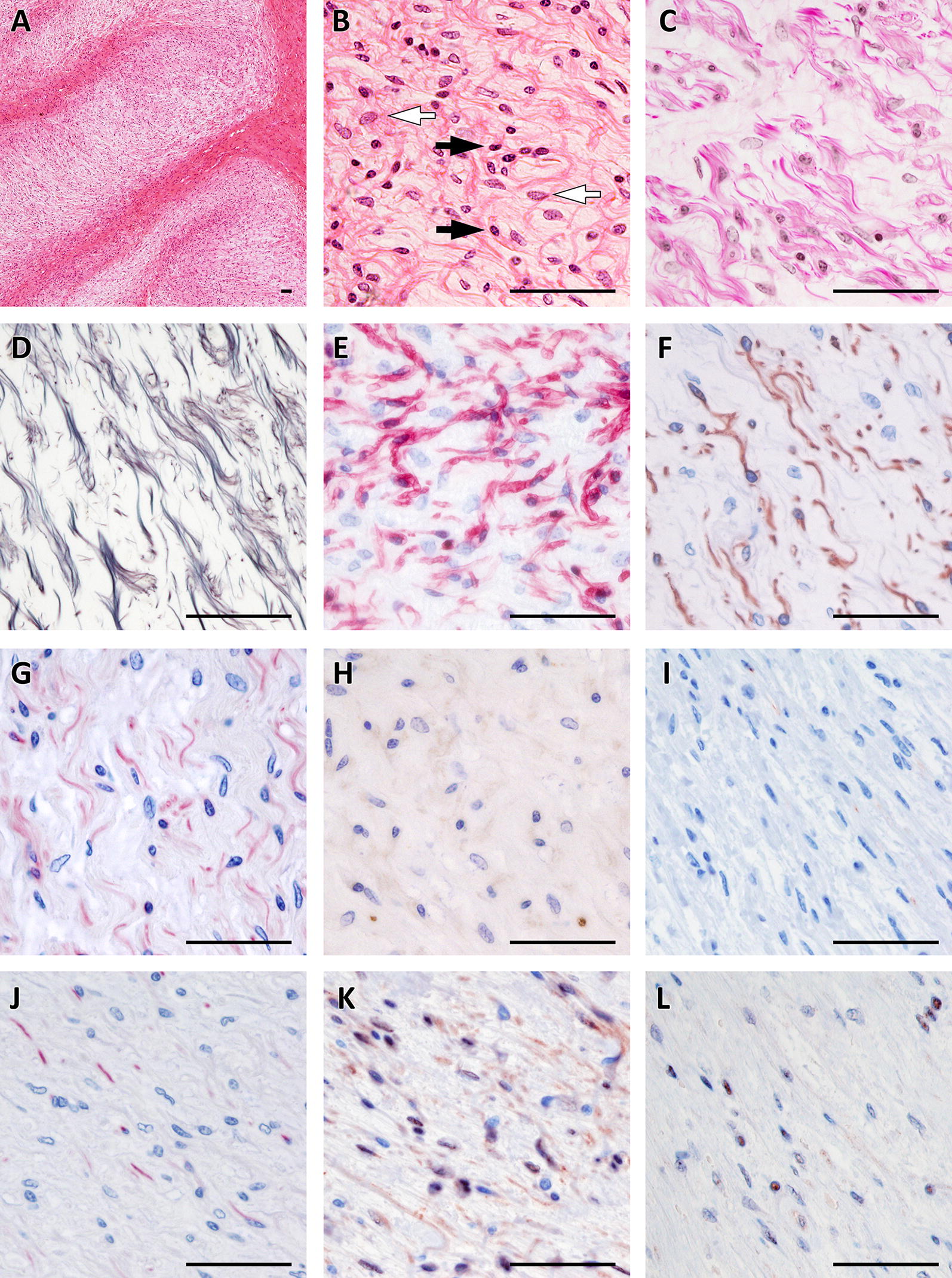
**Neurofibroma in the bovine celiac ganglion**. Myxoid parts with comparatively few cells in bovine neurofibroma of the celiac ganglion can be distinguished at 40 × magnification (**A** Hematoxylin/Eosin). A loss of structure with two types of dominating cell populations can be seen at 400x magnification: (1) small elongated nuclei with condensed chromatin (Schwann cells, black arrows) and (2) large rather ovoid nuclei with coarse chromatin (fibroblasts, white arrows) (**B**). Elastica-van-Gieson staining reveals teared-looking collagen fibres reminiscient of “shredded carrots” (**C**) while Gomori staining shows tattered remains of the endoneuria basal membranes of Schwann cells. Schwann cells in the tumor are positive for S100 (**E**), Vimentin (**F**) and at a low level GFAP (**G**). Few axons positive for Neurofilament (**J**) and PGP9.5 (**I**) are visible and occasionally MPB-positive ones (**H**). Schwann cells express Neurofibromin 1 and 2 in their nuclei (**K**, **L**). bar = 50 µm.

Tumors varied regarding their density of cell nuclei and structure. In some, spare cells seemed to be embedded in a myxoid matrix (Figures  1A, B) while others had a higher cell count in a stromal matrix (Figures  1E, F). Occasionally, the tumor cells seemed to form thin layers beneath a pseudo capsule (Figure 1G). Cells tended to grow in a storiform pattern, but also infiltrated nerve tissue (Figures 1C, D). In Figures  2 and 3, several features of the described cattle neoplasms were compared to regular peripheral nerve tissue. Loosely woven reticular fibers, as produced by Schwann cells and fibroblasts, as well as a loss of the perineurium were visualized by Gomori silver impregnation in all tumors (Figure 2D in contrast to 3D). Elastica-van-Gieson staining revealed an elevated content of collagen fibers in tumor tissue (Figure 3C), resembling “shredded carrots”, a typical feature of neurofibroma. We found both two prevalent cell populations to be without discernible margins. Those two cell types had the morphologic appearance of (1) Schwann cells, i.e. cells with small elongated nuclei and condensed chromatin (Figures  1B, H and 3B; black arrows) and (2) fibroblasts, i.e. cells with large, rather ovoid nuclei and coarse chromatin (Figures  1B, H, and 3B, white arrows). Mitotic figures were rare in all tumors (less than 1 mitosis in 10 high power fields of 400 × magnification) and none of them had necrotic areas, indicating benignity. Immunohistochemistry confirmed the cell population with small elongated nuclei and condensed chromatin to be Schwann cells as this cell population stained positive for S100, Vimentin and sometimes weakly for GFAP (Figures  3E, F and G). None of the tumor cells stained positive for PGP9.5. Single intact nerve fibers drawing through the ganglion were seen and stained positive for PGP9.5, Neurofilament and occasionally MBP (Figures  3H, I, J; for comparison Figures  2H, I, J). Taken together, though different in density of cell nuclei and structure, all tumors in the celiac ganglion could be diagnosed to be neurofibroma, a *per definitionem* benign peripheral nerve sheath tumor mainly consisting of Schwann cells and fibroblasts and sometimes perineural cells. Further markers for non-myelinating Schwann cells like the expression of p75^NTR^ and/or myelinating Schwann cells such as periaxin were not considered necessary for the diagnosis.

In a next step, we wanted to exclude a loss of function of the tumor suppressor proteins neurofibromin 1 and neurofibromin 2, therefore immunostainings against the respective proteins were applied to all tumor sections. Neurofibromin 1 and 2 are involved into the pathogenesis of neurofibromatosis 1, neurofibromatosis 2, and schwannomatosis in humans. Both proteins were detectable in the present neurofibroma (Figures  3K, L). Neurofibromin 1 was visible in the cytoplasm and nucleus while neurofibromin 2 staining was confined to the nucleus.

### Neurofibroma in the celiac ganglion: frequency of occurrence

**Figure 4:**
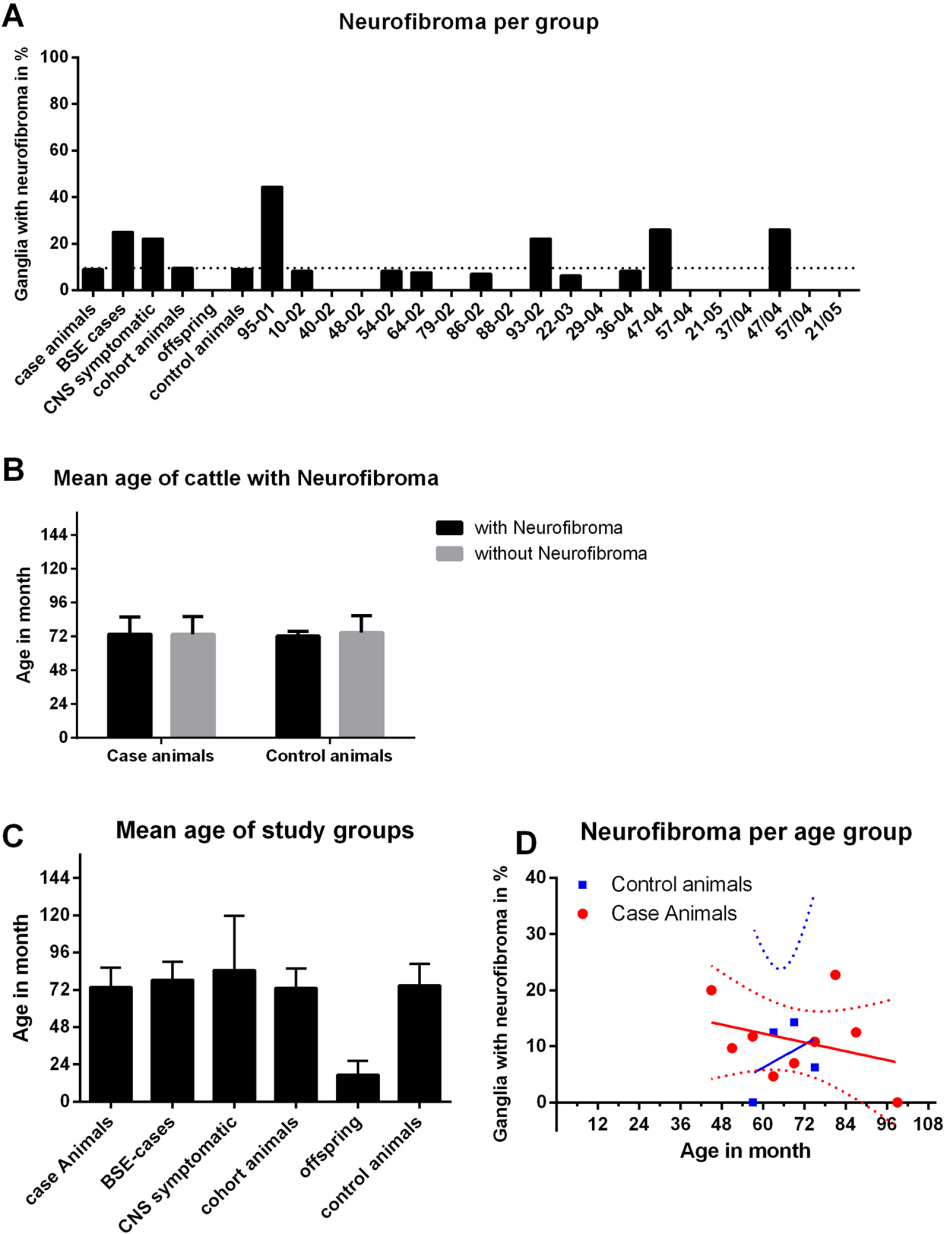
**Frequency of neurofibroma in the different cattle group**s. The percentage of animals with neurofibroma in the celiac ganglion is depicted for each group (**A**) with the dotted line showing the average frequency of neurofibroma for the cohort cattle (9.63%). There was no significant difference in age between case animals with or without neurofibroma in the celiac ganglion and control cattle with or without neurofibroma (**B**). The only study group with obviously younger animals was the offspring of BSE animals (**C**). Regression analysis for a correlation between increasing age and the occurrence of neurofibroma is depicted for case animals (red) and controls (blue). Case animals have a determination coefficient of r^2^ = 0.1047 and a probability of error of *p* = 0.3893. Control animals have a determination coefficient of r^2^ = 0.166 and a probability of error of *p* = 0.5918 (**D**). There is no correlation between age and occurrence of neurofibroma detectable in these groups.

For all study groups, the frequency of neurofibroma in animals with analyzable ganglia celiaca is given in Table 4 (in addition to the numbers and the age of all animals and animals with analyzable celiac ganglia). Available ganglia in the BSE group only sum up to four cattle. We decided to group cohort cattle and BSE animals into the group “case animals”, since they have been estimated to harbor a similar risk for prion disease in the original study set-up. In the case animal group neurofibroma in the celiac ganglion occurred with an incidence of 9.91%. No animals in the offspring group with 25 available ganglia had a detectable neurofibroma in the celiac ganglion. For all cohort animals 9.63% of the investigated celiac ganglia were found to have neurofibroma, however, their number varied between the single cohorts from zero to 44.4% (i.e. cohort 95/01, Figure 4A). In the control group 9.09% of the animals were found to have neurofibroma in their celiac ganglion (Figure 4A and Table 4).

To investigate a possible correlation between tumor incidence and increasing age, the frequency of neurofibroma in age groups (for case animals in red and controls cattle in blue) spanning 6 months each is plotted in Figure 4D. For both, case animals and control cattle, the graphical presentation of the data does not indicate a higher frequency of neurofibroma with increasing age in this comparatively small age span in the samples. Case animals have a determination coefficient of r^2^ = 0.1047 and a probability of error of *p* = 0.3893 and control animals have a determination coefficient of r^2^ = 0.166 and a probability of error of *p* = 0.5918.

## Discussion

Twenty-two of 222 (9.91%) BSE related Holstein Frisian cattle (BSE animals and their cohorts) and six of 66 (9.09%) control cattle designated for human consumption had histopathologically detectable neurofibroma in their celiac ganglion. In the completely unrelated group of CNS symptomatic animals two out of nine (22.22%) cattle had neurofibroma in the celiac ganglion. Unfortunately, the number of examined celiac ganglia from actual BSE cattle is with four animals even smaller, since the gastrointestinal tract (in contrast to the carcass) had already been disposed of by the time that a cow was tested positive for BSE.

The culling of otherwise healthy cattle, i.e. the BSE cohort animals, offered the unique possibility to examine tissues from healthy dairy cattle regarding physiological and pathological changes. Still, it has to be kept in mind that these animals do not represent a random sample of the cattle population in Germany. Control animals were matched in age, breed and region with the cohort cattle, and were considered to be healthy and safe for human consumption. Of course, subclinical/underlying health issues like problems in fertility, udder or feet, might have been reasons for slaughter for the latter, but there does not seem to be a relationship between neurofibroma and any of these factors since tumor frequency does not differ significantly between cohort- and control animals. Even more important about the observation that case animals and cohort animals have a similar percentage of animals with neurofibroma is the conclusion that there seems to be no relationship between the occurrence of neurofibroma and bovine spongiform encephalopathy. The BSE cohort animals are at a greater risk to harbor prion disease themselves than the control animals. Obviously, this higher risk is not correlated with the occurrence of neurofibroma in the celiac ganglion, even though the infectious agent is passing this ganglion on its way to the central nervous system in BSE [3, 5].

Due to the parameters the controls were selected by (age, breed and district of cohort animals), they are not a representative sample for the cattle population in Lower Saxony in Germany either. The control cattle provide an impression, however, of what to expect in Holstein–Friesian cattle with a mean age of 74 month in the North of Germany, where this is the prevailing breed. The CNS symptomatic group consists of animals that displayed neurological signs, but were not related to BSE animals or their cohorts. In view of the group size (*n* = 9) the higher percentage of neurofibroma in the celiac ganglion is not significant.

Among the domestic animals, cattle seem to have benign peripheral nerve sheath tumors more often than other species [13]. Bovine benign peripheral nerve sheath tumors found in abattoirs or during targeted dissections are usually macroscopic findings in ganglia of the heart, paravertebral ganglia of thorax and mediastinum, intercostal nerves and parts of the plexus brachialis [10–12]. The careful attention paid to the pluck is likely to play a role for the frequent observation of macroscopic tumors in these locations. An Australian study also observed benign peripheral nerve sheath tumors in the cervical cranial ganglion and plexus hepaticus [11]. Bovine benign peripheral nerve sheath tumors have also been reported in the trigeminal nerve [21]. It must be noted that the original setting of this study excluded the sampling of thoracic ganglia. So far, only one case report has been published that assumes a benign peripheral nerve sheath tumor in the bovine celiac ganglion [22] since the celiac ganglion was enlarged and the animal already had benign peripheral nerve sheath tumors in the brachial plexus, several cerebral and intercostal nerves, and the cervicothoracic ganglion. Unfortunately, in this case the celiac ganglion was not examined by histopathology. Therefore, our study is the first to provide evidence of neurofibroma in the bovine celiac ganglion due to a thorough investigation in a rather large sample of Holstein Frisian cattle. Without the systematic histologic attempt to find present disease-associated prion protein in several ganglia, neurofibroma in the celiac ganglion would have been overlooked.

The fact that benign peripheral nerve sheath tumors were previously hardly ever reported in the celiac ganglion of cattle is certainly owed in part to its rather poor accessibility in the slaughtering process. It is usually neither inspected macroscopically nor palpated during meat inspection. But even if it was palpated on a regular basis most neoplastic changes would escape the investigator. Small neoplasms are presumably more easily missed in this rather large ganglion than in ganglia of the thorax. In our study, a celiac ganglion seldom made the appearance of being enlarged during the tissue preparation for histology. Interestingly, the celiac ganglion was the only one with neoplasia when compared to the other three examined ganglia. As no more spinal ganglia or the brachial plexus were examined, the occurrence of multiple neurofibroma cannot be completely ruled out. Still, with the trigeminal ganglion, the proximal ganglion of the vagus nerve and the cervical cranial ganglion, two ganglia of cerebral nerves plus a second sympathetic ganglion have been investigated and found to be devoid of neoplasia of any kind.

The general occurrence of neoplasia in central and peripheral nerve tissues of the cattle investigated here largely confirm the results of previously published studies: Within the framework of the original study design also defined brain regions of each animal were examined and in none of the asymptomatic cattle neoplasia were found. In the CNS symptomatic group, however, one of the nine cattle (the only Holstein- German Simmental crossbreed) had a malignant peripheral nerve sheath tumor of the vagal nerve with rhabdoid differentiation, which was located in the cerebello-pontine angle [9]. In other histopathologic studies, the finding of brain tumors in cattle is usually linked to neurologic symptoms and neoplasia of the brain are reported with a frequency of ~2–7% [6–8, 23]. Even more, the presence of neurofibroma in the celiac ganglia in 9.09% of the control cattle and 9.91% of the case animals, both of the groups being seemingly asymptomatic comes as a surprise and raises the question if they are as unproblematic as they seem to be at first sight.

As mentioned before, benign peripheral nerve sheath tumors, frequent as they are as incidental findings in cattle compared to other domestic animals, do not cause symptoms in most cases [10–12]; but their growth may lead to a loss of function of the affected nerve [24]. The celiac ganglion innervates directly or via cross-links reticulorumen, omasum and abomasum as well as wide intestinal segments. It is involved in the gastrointestinal motility and sphincter contraction [25]. Malfunction may cause disrupted motility and absorption; so clinical symptoms could reach from diarrhea to obstipation, culminating in motility disorders like abomasal displacement [26]. For abomasal displacement a genetic component seems to exist [27], although the event is usually triggered by other factors. It is possible that neurofibroma in the celiac ganglion of Holstein Frisian cattle increase the incidence of abomasal displacement in this breed by increasing the risk of motility disorders. However, this hypothesis is difficult to prove since the celiac ganglion can only be examined thoroughly post-mortem. Many cattle sent to the abattoir with abomasal displacement in their antecedent would need to be sampled by a person trained to extract the remains of the celiac ganglion from the gut.

In a more recent study, peripheral nerve sheath tumors were found with a frequency of 3% among older cattle (~11 years) in Danish abattoirs [15]. Although the animals investigated in our study were younger than the ones found to have peripheral nerve sheath tumors in the Danish study [14, 15], we found ~9-10% of the German case animals (*n* = 222, mean age 73.4 months or 6.1 years) and control cattle (*n* = 66, mean age 74.4 months or 6.2 years) to have neurofibroma in the celiac ganglion. Animals with a similar age in the Danish study (between 6 and 7 years old) had peripheral nerve sheath tumors with a frequency below 0.5%. It must be kept in mind though, that any neoplasms in the Danish study were initially detected mascroscopically and not by histological examination. The subsequent histopathological examination of the bovine peripheral nerve sheath tumors showed most of them to be benign, i.e. schwannoma, neurofibroma or a hybrid of both (perineurioma was not detected). Only a small fraction of the tumors could be classified to be malignant peripheral nerve sheath tumors by various criteria of malignancy [14]. In our study, age between animals with and without neoplasms in the case animal group and control group varies only slightly and the neurofibroma per group linear regression analysis does not offer a significant correlation between age and the occurrence of neurofibroma (see Figure 4). Presumably, the latter would be different if the age span was larger than the cohort animals and their controls admit due to their selection parameters determined by the original BSE cases in the North of Germany. The fact that the BSE offspring, with their significantly lower age (*n* = 25, mean age 17.3 months or 1.4 years) in comparison to all other study groups, do not have neurofibroma is the only hint in this study that the occurrence of neurofibroma is linked to a certain age.

When viewed separately, huge differences between the single cohorts exist regarding the frequency of occurrence of neurofibroma. The great variation between zero animals in certain cohorts to 44.4%, i.e. in cohort 95/01, clearly argues in favor of genetic components since most farmers tend to breed and keep their own females for milk production. The genetic aspect could be inspected more closely by analyzing pedigrees of cows with neurofibroma to identify sires that might foster an elevated risk for benign peripheral nerve sheath tumors. The Danish study identified four Holstein bulls whose offspring appear to have an increased risk for developing benign peripheral nerve sheath tumors [15]. Twenty-eight affected animals and 28 unaffected animals of the Danish study also underwent a genome wide association study (GWAS) which identified a single nucleotid polymorphism at chromosome 27 to be associated with peripheral nerve sheath tumors, but the authors interpret it with caution since the small number of animals provides little power. This would be similar for a GWAS of the 30 affected animals (22 case animals, six controls and two CNS symptomatic cattle) of the present study on its own.

Genetic cases of cutaneous neurofibromatosis in cattle were described 40 years ago in Slovakia where more than 50 calves developed neurofibroma a few weeks after birth; the event was linked to a fusion of chromosome 1 and 29 [28]. Human Neurofibromatosis type 1 is caused by a loss of function of neurofibromin 1, which supposedly functions as a tumor suppressor gene, and leads to multiple cutaneous neurofibroma. Approximatively 50% of neurofibromin 1 mutations occur spontaneously while the other 50% are inherited. Neurofibromatosis type 2 is a hereditary autosomal dominant disease that leads to multiple (often bilateral acustic) schwannomas due to a loss of function of neurofibromin 2 (Merlin) [29]. Schwannomatosis itself also causes multiple schwannomas, but the reason here seems to be mutations of the SMARCB1 and LZTR1 genes, though frequently accompanied by a loss of neurofibromin 2 [30]. Cutaneous neurofibromatosis in adult cattle with similarities to human neurofibromatosis 1 upon characterization has been described [31], albeit in few animals. Since in our study animals had neurofibroma only in the celiac ganglion, a different tumor genesis seems to be more likely that is linked to increased age as found in the Danish Holstein cattle. Yet, to make mutations of neurofibromin 1 and neurofibromin 2 less probable we demonstrated the presence of both resulting proteins in neurofibroma of the celiac ganglia by immunohistochemistry. Hence, we assume that these genes are not affected by mutations while, of course, immunohistochemistry only provides evidence of an intact binding site of the proteins. Mutations of other, so far not identified, genes cannot be ruled out and would require further investigations as discussed above.

## Data Availability

The presented data are part of the thesis work of Insa Dammann. They are available after finalization of the thesis and on reasonable request.
