# Correction to: Unexpected high frequency of neurofibroma in the celiac ganglion of German cattle

**DOI:** 10.1186/s13567-020-00855-0

**Published:** 2020-10-15

**Authors:** Insa Dammann, Wiebke M. Wemheuer, Arne Wrede, Wilhelm E. Wemheuer, Amely Campe, Jutta Petschenka, Ulf Schulze-Sturm, Uwe Hahmann, Claus P. Czerny, Pia Münster, Bertram Brenig, Lothar Kreienbrock, Christiane Herden, Walter J. Schulz-Schaeffer

**Affiliations:** 1 Institute of Neuropathology, Medical Faculty of the Saarland University, Homburg, Germany; 2 Institute of Neuropathology, University Medical Center Goettingen, Göttingen, Germany; 3 Institute of Veterinary Medicine, University of Goettingen, Göttingen, Germany; 4 Department for Biometry, Epidemiology and Information Processing (IBEI), University of Veterinary Medicine and WHO-Collaboration Centre for Research and Training at the Human-Animal-Environmental Interface, Hannover, Germany; 5 Institute of Pathology, Veterinary Faculty, Justus Liebig University, Gießen, Germany; 6 Landeslabor Schleswig–Holstein, Geschäftsbereich 2 Veterinärwesen, Neumünster, Germany; 7 Boehringer Ingelheim Pharma GmbH & Co. KG, Cancer Immunology & Immune Modulation, Biberach an der Riss, Germany; 8 Department of Paediatrics, University Medical Centre Hamburg-Eppendorf (UKE), Hamburg, Germany; 9 Elanco Deutschland GmbH, Hauptsitz Werner-Reimers-Str. 2-4, Bad Homburg, Germany

## Correction to: Vet Res (2020) 51:82 10.1186/s13567-020-00800-1

In the original publication of this article [1], the authors identified an error in the author name of Bertram Brenig.

The incorrect author name is: Bertram Brening.

The correct author name is: Bertram Brenig.
